# The Relationship between Anemia and Helicobacter Pylori Infection in Children

**DOI:** 10.3390/children9091324

**Published:** 2022-08-30

**Authors:** Ancuta Lupu, Ingrith Crenguta Miron, Anca Lavinia Cianga, Andrei Tudor Cernomaz, Vasile Valeriu Lupu, Dragos Munteanu, Dragos Catalin Ghica, Silvia Fotea

**Affiliations:** 1 Pediatrics Department, “Grigore T. Popa” University of Medicine and Pharmacy, 700115 Iasi, Romania; 2 III-rd Medical Department, “Grigore T. Popa” University of Medicine and Pharmacy, 700115 Iasi, Romania; 3 I-st Medical Department, “Grigore T. Popa” University of Medicine and Pharmacy, 700115 Iasi, Romania; 4 Preventive Medicine, “Grigore T. Popa” University of Medicine and Pharmacy, 700115 Iasi, Romania; 5 Pediatrics Department, Faculty of Medicine and Pharmacy, “Dunarea de Jos” University of Galati, 800008 Galati, Romania

**Keywords:** anemia, *Helicobacter pylori*, iron deficiency, children

## Abstract

(1) Background: Many studies suggest that *Helicobacter pylori* (*H. pylori*) infection is associated with a higher prevalence of anemia. The aim of this study is to explore this fact for a pediatric population from the northeast of Romania; (2) Methods: A correlational retrospective study between infection with *H. pylori* and anemia was performed on a group of 542 children in a pediatric gastroenterology regional center in Northeast Romania; (3) Results: Out of 542 children with confirmed *H. pylori* infection, microcytic hypochromic anemia was present in 48 children, of whom 7 (14.5%) also had iron deficiency.; (4) Conclusions: The study results demonstrate a significant association of *H. pylori* infection with iron-deficiency anemia and iron deficiency in children in accordance with the results established in the published literature. Although the direct relationship between them it is not clear yet, prevention represents one of the first clinical measures that need to be implemented when encountering a refractory moderate to severe iron-deficiency anemia and, especially, when associated with gastrointestinal tract symptoms.

## 1. Introduction

*H. pylori* is a spiral-shaped pathogenic bacterium located on the human gastric mucosa, which infects about half of the world’s population and is the main etiological factor for peptic ulcer disease and gastric malignancy [1]. The infection causes disease predominantly in adults, while children do not manifest any specific symptoms [2] and peptic ulcer disease is rare enough in childhood [3]. 

*H. pylori*-associated inflammation represents a risk factor for other gastrointestinal diseases including gastroesophageal reflux disease and duodenal ulcers and its persistence within the host’s stomach causes chronic inflammation which may lead to gastric mucosa-associated lymphoid tissue (MALT) lymphoma, gastric cancer and adenocarcinoma. Many studies have also indicated definitive treatment of early MALT lymphoma after complete eradication of H. *pylori* infection [4].

Moreover, *H. pylori* infection is associated with multiple extra-digestive infections and diseases from the autoimmune spectrum, cardiovascular diseases, pancreatic and colonic diseases, hepato-biliary system diseases, bronchiectasis, diabetes mellitus, neurological diseases and hematological diseases, thus complicating case management for these patients [5].

Several diagnostic procedures are available in order to establish the diagnostic which include non-invasive and invasive tests. Non-invasive tests include ureolytic-based tests, serum bicarbonate and ammonia vapor tests, immunological techniques (serological tests, salivary and urinary tests, near-patient assays) and the stool antigen test, having the advantage of being more acceptable and more comfortable for the patients [6]. On the other hand, invasive tests or endoscopic techniques along with an appropriate specimen increase the accuracy of the results [7].

Yuan et al. describe in their latest meta-analysis the importance of *H. pylori* infection among the pediatric population, emphasizing its high prevalence in this age group and the relationship of causality between the infection and lower economic status, parents or siblings infected with *H. pylori,* lack of access to a sewage system and drinking unboiled or untreated water [8].

Studies in the literature have reported that *H. pylori* infection could be a potential cause of iron-deficiency anemia (IDA). IDA represents a global public health problem which has a significant impact on human health and social and economic development. Inadequate iron intake, chronic blood loss and impaired iron absorption are among the causes of IDA [9].

There are many studies that describe an association between *H. pylori* infection and IDA, but the biological explanation for *H. pylori* infection causing iron-deficiency anemia remains unknown. Initially, sideropenic anemia was considered to be caused by occult blood loss due to chronic superficial active gastritis caused by *H. pylori* [10], but subsequent studies did not confirm this theory [11]. However, *H. pylori* infection can cause disorders in iron assimilation and increased iron requirements. Hypoacidity caused by pangastritis and a low level of ascorbic acid in the stomach of patients infected with *H. pylori* may affect the absorption of iron in the duodenum [12]. In addition, levels of lactoferrin gastric mucosa (an iron-binding protein) are high in patients infected with iron-deficient *H. pylori*, showing a possible role between increased lactoferrin sequestration and iron utilization by the body [13]. *H. pylori* also competes with the host for available food grade iron. *H. pylori* has several iron acquisition systems, which can capture iron available in the microenvironment of the stomach lumen [14]. Moreover, there are studies that indicate that an iron-deficiency anemia which does not respond to iron therapy can be resolved by eradicating *H. pylori* from the stomach [15].

Although the data regarding the association between *H. pylori* infection and anemia has been well-documented for a long time, we aim to present the results we obtained in the northeast of Romania and to compare our numbers to the preexisting ones.

## 2. Materials and Methods

A retrospective study was carried out over a period of 3 years (1 April 2013–31 March 2016), on a group of 1757 patients of both sexes, mainly hospitalized in the Gastroenterology Pediatric Clinic, but also in the other clinics of the Emergency Hospital for Children "St. Mary" in Iasi, Romania, admitted for upper digestive tract complaints. A correlational study between those infected with *H. pylori* (542) and anemia was conducted. 

Available endoscopy and *H. pylori* testing data were used as main inclusion criteria; patients with previous therapy to eradicate *H. pylori*, simultaneous consumption of aspirin and non-steroidal anti-inflammatory drugs, patients with esophageal stricture or esophagitis secondary to systemic diseases, with active gastrointestinal bleeding, or any past history of esophageal or gastric surgery and previous known anemias were excluded.

Given the existing data in the literature regarding the association of *H. pylori* infection and sideropenic anemia in children, we explored the following parameters: complete blood count (CBC) with platelet count and hemoglobin value and erythrocyte indices useful in classifying anemias (MCV, MCH, MCHC) and serum iron. 

Anemia was defined according to the World Health Organization (WHO) age criteria; that is, hemoglobin level of <11 g/dL (110 g/L) in children <5 years, <11.5 g/dL (115 g/L) in children with ages between 5 and 12 years and <12 g/dL (120 g/L) for patients with ages between 12 and 18 years old [16].

### 2.1. Endoscopy

Upper gastrointestinal endoscopy was performed on all patients using Pentax and Olympus video pediatric endoscopes to observe the macroscopic aspect and take the biopsies. Intravenous sedation was given; for children aged below 10 years, general anesthesia was performed. 

Biopsies taken from the gastric antrum and corpus were used for rapid urease testing and for bacteriological and histological examination. All 542 patients had *H. pylori* infection diagnosed by the urease test.

The research was based on the accumulation of data from patient observation sheets and discharge notes in the hospital database files with endoscopy results.

### 2.2. Statistical Analysis

The data regarding the study group were organized in a tabular structure containing a number of 90 categorical variables and 2 continuous variables.

The processing of this data was carried out using the SPSS 17.0 platform and the Microsoft Excel software.

The study group was stratified according to different parameters—the significance of the differences was assessed using non-parametric chi-square tests (taking into account the large number of categorical variables). 

The extrapolation of the results obtained to the general population should be made with caution given that the data came from the analysis of a batch (probably influenced by a number of factors—some obvious, represented by seasonality, accessibility) and not a representative sample.

## 3. Results

*H. pylori* infection in children has been associated with various extra gastric pathologies: iron-deficiency anemia, growth retardation, chronic idiopathic thrombocytopenic purpura and diabetes. The suggested role of *H. pylori* in the pathogenesis of extraintestinal manifestations is assumed on the following considerations: local inflammation has systemic effects, gastritis with *H. pylori* is a chronic process that can last for years, persistent infection leads to a chronic inflammatory and immune response which is capable of inducing lesions locally and at a distance from the main site of infection [17,18,19].

From the 1757 (68.9% females) pediatric patients (mean age 13.1 +/− 3.5, range 1–18) with digestive complaints admitted to our unit, 542 were diagnosed with *H. pylori* infection on the base of positive urease tests.

Of them, microcytic hypochromic anemia was present in 48 children, of whom 7 (14.5%) also had hyposideremia (Table 1). From the statistical analysis, it was observed that there was a very strong significant association between *H. pylori* infection and iron deficiency and iron-deficiency anemia (χ2; *p* < 0.001).

Regarding gender, there were 10 boys and 38 girls with iron-deficiency anemia in the *H. pylori*-positive group compared to 29 boys and 67 girls in the *H. pylori*-negative group with no significant association.

Considering the existing data in the literature regarding the association of H. *pylori* infection in children with immune thrombocytopenic purpura, we evaluated the variations of the platelet counts in our patients, but only one case of thrombocytopenia was registered. Therefore, from the statistical analysis, we did not obtain a significant association between *H. pylori* infection and low platelet count (χ^2^; *p* > 0.05).

No significant association between anemia and *H. pylori* status was found (chi squared) for various age subgroups (Table 2).

There were no significant differences in terms of anemia between subgroups stratified using gender, age group or both (Table 3).

Besides the known causes of iron-deficiency anemia, in the last twenty years, an association has been established between *H. pylori* and iron-deficiency anemia in pediatric age groups [20,21,22]. The proof of the relationship between iron-deficiency anemia and *H. pylori* infection in children is the beneficial effects of *H. pylori* eradication on pre-existing iron-deficiency anemia which were evaluated in a few studies (Table 4) [23].

## 4. Discussion

In this study, we assessed the association between *H. pylori* infection and iron deficiency or iron-deficiency anemia among patients attending our Gastroenterology Pediatric Clinic from Iasi, in the Northeast Romania, an area with high prevalence of *H. pylori* infection.

The results of our study were obtained from an area which has the highest incidence of rural poverty in the European Union. A recent review describes that in Europe, the highest *H. pylori* infection rates were found in Eastern and Southern Europe which are also the regions with the most important gastric cancer incidence rate in the European Union [28]. 

Unfortunately, in Romania there is a lack of epidemiological data that evaluate the prevalence of *H. pylori* among children. However, Corojan et al. observed in their study a decline of prevalence of the infection in Northwest Romania over a 30-year interval, suggesting the importance of easier access to medical services and a perpetual improvement of the socio-economic conditions [29].

Unarguably, the socio-economic and ethnic backgrounds of our subjects, such as: many family members and crowded living conditions, poor sanitation and lack of clean water represent an important factor risk for the evolution of *H. pylori* infection.

Our study group showed a significant increase in *H. pylori* cases in older patients—this may possibly be explained by cumulating exposure over time and similar patterns have been reported for developing countries [30].

The higher prevalence of *H. pylori* infection for females has also been previously reported; perhaps, lower gastric acidity may play a role [31].

Our results are consistent with the findings of Bille et al. who describe a high prevalence of IDA with microcytosis and hypochromia among the pediatric population examined recently in their study in Cameroon [32]. 

By examining the relationship between *H. pylori* infection and serum iron levels in a study conducted on 300 patients in Pakistan, Kishore et al also found that the mean serum iron level was significantly lower in participants with *H. pylori* infection compared to those who did not have *H. pylori* infection (110.72 ± 28.38 μg/dL vs. 162.5 ± 21.18 μg/dL; *p*-value: <0.0001) [33].

Extra-digestive tract conditions such as iron deficiency or IDA have been increasingly associated with the *H. pylori* infection [32]. Moreover, many studies proved that the anemia that did not respond to iron therapy was resolved after eradication treatment of *H. pylori*, indicating a possible impact of the bacteria in the iron metabolism [34,35].

For example, in their study on 21 adolescents with iron-deficiency anemia, Fotia et al. proved the presence of infection with *H. pylori* in 61% of patients and three months after the eradication of the bacteria using triple therapy, the value of the hemoglobin increased on average by 2 g% [36].

Similar findings were described by Tanous et al. in their very recent study where they noticed an important improvement in hemoglobin value and ferritin concentrations as well as a rate of 60% of iron normalization after complete eradication of *H. pylori* [37].

Clearly, more studies and clinical observations need to be carried out in order to explain the causality between IDA and *H. pylori* infection.

It is well known that *H. pylori* infection and IDA are prevalent in disadvantaged global populations [38] and we confirmed the same information for the pediatric population evaluated in the northeast of Romania. 

In our study performed on 542 patients with *H. pylori*, we found that microcytic hypochromic anemia was present in 48 children, of whom 7 (14.5%) also presented with hyposideremia. From the statistical analysis, it was observed that there was a very strong association between *H. pylori* infection and iron deficiency and iron-deficiency anemia (χ^2^; *p* < 0.001).

Our results are consistent with the data described by Qu et al in their meta-analysis which comprised a total of 15,183 patients from 20 studies where the association between *H. pylori* and IDA was demonstrated and, moreover, showed that treating *H. pylori* infection can improve hemoglobin and serum iron levels [39].

On the other hand, in their case-control study conducted on 100 children with *H. pylori* infection and 109 children without infection, Haghi-Ashtiani et al. did not find any associations with IDA in children [40]. The same idea was described by Hou et al who did not find any significant relationship between H. *pylori* infection and serum iron levels, thus emphasizing the need for further studies of different strains of *H. pylori*, posttreatment measurement of serum iron and ferritin and the socioeconomic status of the pediatric population evaluated [41].

## 5. Conclusions

In conclusion, the results of this study demonstrate significant association of *H. pylori* infection with iron-deficiency anemia and iron deficiency in children in accordance with the results established in the published literature.

Although the direct relationship between them it is not clear yet, prevention represents one of the first clinical measures that need to be implemented when encountering a refractory moderate to severe iron-deficiency anemia and, especially, when associated with gastrointestinal tract symptoms.

## Figures and Tables

**Table 1:** Structure of the group of children with *H. pylori* infection and iron-deficiency anemia.

HP (+)		Hyposideremia (−)	Hyposideremia (+)	Total
**Hypochromic microcytic anemia**	(−)	482	12	**494**
	(+)	41	7	**48**
	**Total**	**523**	**19**	**542**

Fisher’s exact probability <0.001 (2-sided).

**Table 2:** *H. pylori* infection and anemia within various age groups.

Age Group	IDA	*H. pylori* Negative	*H. pylori* Positive
<3 years	not present	17 (70.8%)	
	present	7 (29.2%)	
4–6 years	not present	63 (75%)	13 (15.5%)
	present	7 (8.3%)	1 (1.2%)
7–10 years	not present	203 (76.9%)	40 (15.1%)
	present	15 (5.7%)	6 (2.2%)
11–13 years	not present	379 (63.7%)	176 (29.6%)
	present	28 (4.7%)	12 (2%)
15–18 years	not present	457 (57.8%)	265 (33.5%)
	present	39 (5%)	29 (3.7%)

**Table 3:** Anemia and gender within various age groups.

Age Group	Anemia	Gender	
		girls	boys
<3 years	not present	6 (25%)	11 (45.8%)
	present	3 (12.5%)	4 (16.6%)
4–6 years	not present	38 (45.2%)	38 (45.2%)
	present	5 (5.9%)	3 (3.5%)
7–10 years	not present	144 (54.5%)	99 (37.5%)
	present	11 (4.1%)	10 (3.7%)
11–13 years	not present	383 (64.3%)	172 (28.9%)
	present	29 (4.8%)	11 (1.8%)
15–18 years	not present	534 (67.5%)	188 (23.7%)
	present	57 (7.2%)	11 (1.3%)
Total	not present	1105 (62.8%)	508 (28.9%)
	present	105 (5.9%)	39 (2.27%)

**Table 4:** Randomized clinical trials of *H. pylori* eradication for iron-deficiency anemia and iron deficiency in children.

Author (Year)/Country	No of Children with IDA/*H. pylori*	Follow-Up Period	Follow-Up	
			IDA	Iron Deficiency
**Choe et al. (1999)/Korea** [24]	**43/25**	8 weeks	↑ Hb with: eradication + iron, eradication + placebo vs. Fe + placebo (*p* = 0.0086)	No significant differences in serum iron or ferritin
**Sarker et al. (2008)/Bangladesh** [25]	260/200	3 months	IDA persistent with: eradication + iron, only eradication, 33%; only iron, 0%; placebo, 45%	Iron deficiency persistence with: eradication + iron, 19%; only eradication, 65%, only iron, 7%; placebo, 78%
**Gessner et al. (2006)/Alaska** [26]	219/219	14 months	IDA persistent with: eradication + iron, 22%; only iron, 14%	Iron deficiency persistence with: eradication + iron, 65%; only iron, 72%
**Fagan et al. (2009)/Alaska** [27]	219/219	40 months	IDA persistent with: eradication + iron, 5%; only iron, 19%	Iron deficiency persistence with: eradication + iron, 52%; only iron, 58%

## Data Availability

The data presented in this study are available on request from the corresponding author.
